# Development of a Gas-Tight Microfluidic System for Raman Sensing of Single Pulmonary Arterial Smooth Muscle Cells Under Normoxic/Hypoxic Conditions

**DOI:** 10.3390/s18103238

**Published:** 2018-09-26

**Authors:** Fenja Knoepp, Joel Wahl, Anders Andersson, Johan Borg, Norbert Weissmann, Kerstin Ramser

**Affiliations:** 1 Excellence-Cluster Cardio-Pulmonary System (ECCPS), Universities of Giessen and Marburg Lung Center (UGMLC), Member of the German Center for Lung Research (DZL), Justus-Liebig University Giessen, D-35392 Giessen, Germany; fenja.knoepp@innere.med.uni-giessen.de (F.K.); norbert.weissmann@innere.med.uni-giessen.de (N.W.); 2 Department of Engineering Sciences and Mathematics, Luleå University of Technology, SE-97187 Luleå, Sweden; joel.wahl@ltu.se (J.W.); anders.g.andersson@ltu.se (A.A.); 3 CMS, Imperial College, London SW7 2AZ, UK; j.borg@imperial.ac.uk

**Keywords:** hypoxia, microfluidic system, Raman spectroscopy, redox reactions on single cell level

## Abstract

Acute hypoxia changes the redox-state of pulmonary arterial smooth muscle cells (PASMCs). This might influence the activity of redox-sensitive voltage-gated K^+^-channels (Kv-channels) whose inhibition initiates hypoxic pulmonary vasoconstriction (HPV). However, the molecular mechanism of how hypoxia—or the subsequent change in the cellular redox-state—inhibits Kv-channels remains elusive. For this purpose, a new multifunctional gas-tight microfluidic system was developed enabling simultaneous single-cell Raman spectroscopic studies (to sense the redox-state under normoxic/hypoxic conditions) and patch-clamp experiments (to study the Kv-channel activity). The performance of the system was tested by optically recording the O_2_-content and taking Raman spectra on murine PASMCs under normoxic/hypoxic conditions or in the presence of H_2_O_2_. Oxygen sensing showed that hypoxic levels in the gas-tight microfluidic system were achieved faster, more stable and significantly lower compared to a conventional open system (1.6 ± 0.2%, respectively 6.7 ± 0.7%, n = 6, *p* < 0.001). Raman spectra revealed that the redistribution of biomarkers (cytochromes, FeS, myoglobin and NADH) under hypoxic/normoxic conditions were improved in the gas-tight microfluidic system (*p*-values from 0.00% to 16.30%) compared to the open system (*p*-value from 0.01% to 98.42%). In conclusion, the new redox sensor holds promise for future experiments that may elucidate the role of Kv-channels during HPV.

## 1. Introduction

Hypoxic pulmonary vasoconstriction (HPV) is a local response of the pulmonary vasculature that diverts blood from poorly to well-oxygenated lung areas. Thereby, HPV maintains optimal arterial oxygenation by matching capillary perfusion to alveolar ventilation on a breath-to-breath basis [1,2]. Consequently, decreased HPV (e.g., during pneumonia, sepsis, anesthesia or liver failure) can lead to life-threatening hypoxemia, whereas prolonged and global alveolar hypoxia (e.g., at high altitudes or in patients suffering from chronic lung diseases) results in exaggerated HPV. This chronic and global vasoconstriction can lead to an irreversible pathological remodeling of the pulmonary vasculature, resulting in pulmonary hypertension (PH) and consequently—due to the constant work-overload—to right heart failure [1,2,3]. Hitherto, the process underlying HPV is not fully understood. It is well established that precapillary pulmonary arterial smooth muscle cells (PASMCs) are the sensor and effector cells in HPV, since they constrict upon exposure to acute hypoxia—thereby narrowing the diameter of resistance pulmonary arteries [1,2,3]. Important signaling molecules that cause the PASMCs to constrict are reactive oxygen species (ROS) and H_2_O_2_ in particular [2]. Recently, the pulmonary-specific isoform 2 of the mitochondrial complex IV subunit 4 (Cox4i2) has been identified as primary oxygen (O_2_)-sensor in PASMCs. O_2_-sensing via Cox4i2 initiates acute HPV by promoting hypoxia-induced ROS-release preferentially at complex III of the electron transport chain [2]. The subsequent change in the cellular redox state is proposed to link O_2_-sensing with PASMC-contraction via inhibition of voltage-dependent K^+^-channels (Kv-channels) that are crucial for mediating membrane potential in PASMCs—thereby controlling Ca^2+^-entry and subsequently vascular tone [4]. Kv-channels are known to be redox-sensitive [4,5,6,7,8], since they are sensitive to H_2_O_2_ [9,10] and changes in both NADP(H) and NAD(H) levels [4,11].

To date, it was impracticable to simultaneously study (a) the activity of Kv-channels in PASMCs and (b) the cellular redox state of the same cell in response to different oxygen-concentrations. For this reason, a gas-tight microfluidic system has been developed that has the option to coincidently perform patch-clamp experiments (to study the Kv-channel activity) as well as Raman spectroscopic investigations on single-cell level (in order to monitor the cellular redox-state) under reversible and tightly controlled normoxic/hypoxic conditions.

For patch-clamp experiments, a micropipette with a narrow tip of less than one micrometer has to be steered towards a cell and attached by application of gentle suction to the cell membrane. This procedure requires careful micromanipulation of the pipette in three dimensions (3D)—which is difficult to achieve in a closed gas-tight system. In addition, hypoxic conditions are usually created in an open system by flooding the cells with hypoxic buffers. By doing so, a level of 5% O_2_ can be reached—which cannot be conserved as truly hypoxic. In a previous study, these technical problems had been solved by maneuvering optically trapped biological cells in a closed system towards a fixed micropipette by moving the xyz-microscope stage in 3D [12]. The system proved to be successful, however, it was (1) intricate to fix the micropipette; (2) the optical steering of the cells emerged to be very time consuming and (3) it was impossible to measure adhesive cells—such as PASMCs. Here, a new design of a closed microfluidic system is presented that overcomes these technical difficulties; a channel opening with a flexible latex glove was added to the closed system. In this channel, the micropipette could be attached and moved like a gearshift in 3D towards the PASMCs that were adhered to the bottom of the microfluidic system.

For monitoring the redox state of cellular biomarkers (cytochrome b, cytochrome c, myoglobin and the vibrational bonds of NADH) within a single PASMC Raman spectroscopy was performed. Spectroscopic investigation on PASMCs are hitherto scare. As stated by Waypa et al., a change in intracellular ROS-production during hypoxia has been detected in PASMCs by using chemiluminescence and DFC fluorescence, whereas electron paramagnetic resonance (EPR) revealed a change in oxidant production [13]. However, the poor resolution of the measurement methods made it unclear whether the ROS levels increased or not. Therefore, Waypa et al. investigated the hypoxia-induced increase in mitochondrial ROS under hypoxic conditions by using redox-sensitive fluorescence resonance energy transfer (FRET) [13]. Their results demonstrated that hypoxia triggers Ca^2+^-influx in PASMCs via augmenting mitochondrial ROS signaling. An essential disadvantage of this method is, however, that the cells need to be stained—meaning that they are not in their native state anymore. Intrinsic NAD(P)H fluorescence has been used to create confocal images by two photon excitation in cardiac myocytes [14]. This method, although promising for studies of metabolic states in living cells, only allows for the study of one biomarker. In contrast, Raman spectroscopy is a powerful tool to investigate mitochondrial biomarkers in native cells, where especially the mitochondrial biomarkers FeS, NADH and cytochrome b and c can be studied simultaneously. For instance, cytochrome c has been monitored during cell apoptosis [15], and also the mitochondrial fitness in yeast cells have been monitored by Raman spectroscopy [16]. Furthermore, the redox state of especially cytochrome c has been investigated in a perfused rat heart [17], living Saccharomyces cerevisiae cells, and isolated cardiomyocytes [18,19]. For this reason, Raman spectroscopy was included in the proposed sensor to monitor the redox state of single PASMCs under normoxic/hypoxic conditions. Here, first tests on the feasibility of Raman spectroscopy were performed without going into detail how individual biomarkers react upon oxygen deprivation.

In the present study, the initial design consideration of the “redox sensor”, as well as modeling of the flows through the microfluidic system, are presented. To functionally test the new gas-tight system, Raman spectroscopy was performed on single primary murine PASMCs under (a) different O_2_ contents, induced by application of a hypoxic buffer or (b) by adding exogenous H_2_O_2_ in a physiological concentration of 124 nM. H_2_O_2_, a common oxidizing agent, was used to test if the observed changes in the Raman spectra in response to hypoxia were due to a change in the cellular redox state. Identical experiments were carried out in a conventional open system to compare the results.

## 2. Materials and Methods

### 2.1. Simulations and Design of the Gas-Tight Microfluidic System

**Figure 1:**
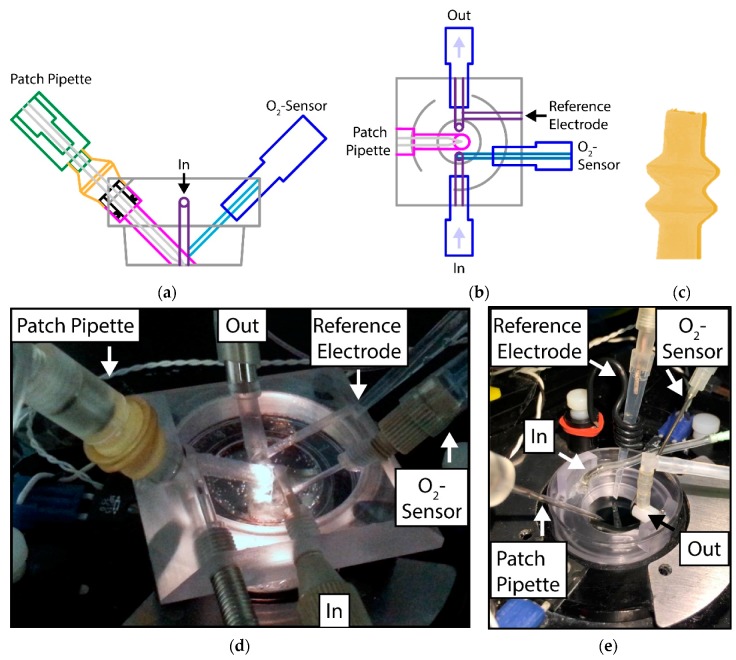
Side view (**a**) and eagle view (**b**) of the CAD drawing of the microfluidic system; (**c**) photograph of the flexible latex glove; (**d**) the closed and (**e**) open system are shown with all components attached. In: Inflow, Out: Outflow.

The design of the microfluidic system drawn in a CAD program was based on (1) the number of entrances and outlets needed (for inflow, outflow, patch-pipette, reference electrode and O_2_-sensor) and (2) the shape of the dishes used for PASMC cultivation. Some views of the CAD drawings are shown in Figure 1a,b.

To describe the fluid flow inside the system, Computational Fluid Dynamics (CFD) were performed using a commercially available software (Ansys CFX 16, Ansys, Canonsburg, PA, USA). For optimization, different outlet geometries based from a simplified version of the CAD design were investigated, since capillary forces in microfluidic systems are increased by internal pressure differences. These capillary forces can lead to a rising of fluids in the in-, and outlets that renders measurements impossible. In order to get optimal conditions, different diameters and shapes of the outlet were tested. In addition, time-dependent simulations were performed to investigate the fluid levels in the measurement section and a Volume of Fluids (VOF) model was implemented to account for both the gas and liquid phase. The fluids in the simulation were defined as Water at 20 °C and Air at 25 °C. The flow rate in the simulations was 5 mL/min and the numerical time-step was set to 10 ms.

**Figure 2:**
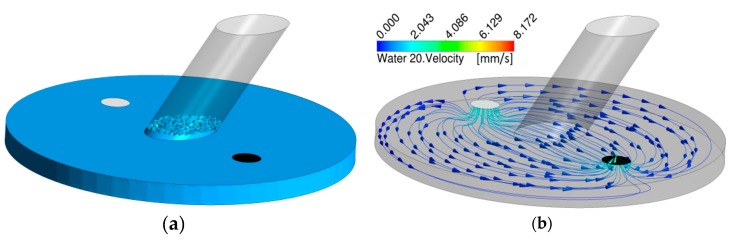
Surface level (**a**) and velocity streamlines (**b**) from inlet (white) to outlet (black) after 100 s.

After simulations with different outlet designs, the diameter was selected to be the same as the inlet diameter (2 mm). This setup gave a quite low fluid build-up in the measurement section which can be seen in Figure 2, together with the velocity streamlines going from the inlet to the outlet after a simulation time of 100 s.

The final base of the microfluidic system was CNC milled in polycarbonate (PC).

A further challenge was to design a gas-tight entrance for the fragile patch-clamp micropipette that allows the careful and precise 3D movement of the pipette. For that reason, a flexible latex glove was constructed by dip-molding: An aluminum mandrel was pre-heated to approximately 60 °C before dipping in a liquid latex resin (Mouldcraft Ltd., Sheffield, UK). No chemical coagulants were used, the heat alone resulted in a sufficiently thick layer of coagulated latex around the mandrel. After extracting the mandrel, it was allowed to dry in air at 100 °C for about an hour. The thickness, and thus the mechanical strength and stiffness of the glove, was adjusted by changing the concentration of the latex resin and the dwell time of the mandrel in the resin. The latex glove, with two bulbs to increase the range of motion, is shown in Figure 1c. In use, the upper end of the glove was stretched over the tip of the pipet holder, whereas the lower end was fitted over a hollow plug, which was sealed to the main body of the flow system using an o-ring. The use of a separate plug, rather than sealing the glove directly to the body of the flow system, allowed the glove to be assembled before the pipet was inserted into the holder, thus reducing the risk of damaging the fragile pipet and making pipet replacement easier. See the assembled flow system in Figure 1d for details.

### 2.2. Experimental Setup for Raman Spectroscopy

A microscope was built by mounting the following items onto a 2′′ post (Thorlabs Inc., Newton, MA, USA);

- a quarz halogen lamp (MI-150, Edmund Optics, Barrington, IL, USA),
- a manual xy-stage (Merzhäuser, Wezlar, Germany),
- a microscope objective holder equipped with a 60× water immersion objective (Olympus, Tokyo, Japan),
- a CCD camera to observe the sample (Guppy, Allied Vision GmbH, Stadtroda Germany),
- two edge filters (532 razor sharp edge and 532 basic edge filters, Semrock, Rochester, NY, USA), both used to guide the laser light onto the sample and to block out the laser light prior to the Raman spectrometer,
- an optical fiber to guide the Raman scattered light into a Raman spectrometer (Shamrock 303i, Andor Technology, Belfast, UK) equipped with an air-cooled CCD camera (Andor Technology, Belfast, UK).

**Figure 3:**
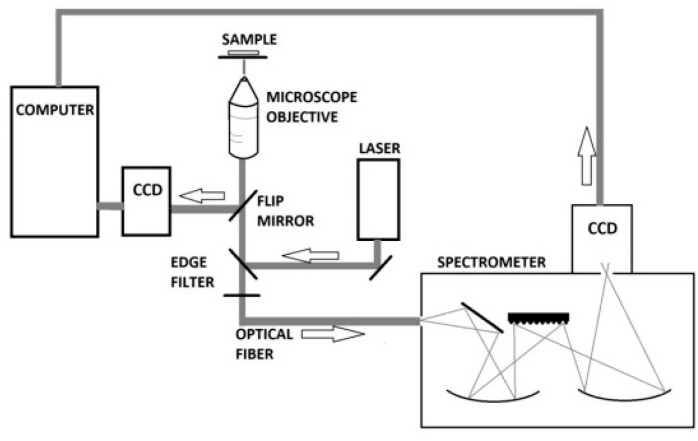
Schematic of the setup, starting with a computer coupled to the spectrometer that was fiber-optically coupled to the microscope.

The final setup is shown in Figure 3.

Raman measurements were carried out with a Shamrock 303i spectrometer and an excitation wavelength of 532 nm (DPSS 532 laser) at an integration time of 120 s at a power of 0.6 mW. The slit into the spectrometer was set to 50 μm giving a spectral resolution of 6 cm^−1^. The laser beam was, in this study, intentionally defocused to 20 μm to average the signal from a large intracellular region from the single PASMC.

### 2.3. Sample Preparation

The isolation of murine PASMCs was approved by the “Institutional Animal Investigation Care and Use Committee” and the appropriate governmental committee. PASMCs from C57BL/6J mice were isolated as previously described [2,20] and grown on glass bottom dishes (35/10 mm, Greiner Bio-One, Frickenhausen, Germany) for 7 to 10 days prior to experiments. During measurements, PASMCs were continuously perfused (0.5 mL/min) with pre-heated (37 °C) Tyrode’s solution (composition in mM: 126.7 NaCl, 5.4 KCl, 1.8 CaCl_2_, 1.05 MgCl_2_, 0.42 NaH_2_PO_4_, 22 NaHCO_3_, 10 Glucose, pH 7.4, 0.5 mL/min, gassed with either normoxic (21% O_2_) or hypoxic (1% O_2_) gas mixture, both containing 5.3% CO_2_, rest N_2_). The glass bottom dish was either (a) kept open (open System, os) by using a perfusion insert (Warner Instruments, Hamden, CT, USA) or (b) covered with the customized microfluidic system (closed system, cs).

### 2.4. Experimental Procedure

Three different types of experiments were carried out for both systems (open vs. closed):

1. In the experiment termed as Redox, the measurement chamber was perfused with normoxic (NOX) or hypoxic (HOX) gas mixture.
2. In the experiment, termed H_2_O_2_, the procedure was repeated by replacing the hypoxic solution with a normoxic solution containing 124 nM H_2_O_2_. Due to its instability, H_2_O_2_ was dissolved directly before application for each individual experiment.
3. In the third experiment—termed as control—the Tyrode’s solution was kept at normoxic O_2_ levels throughout the entire experiment.

The experimental procedure was as follows: First, a Raman spectrum under normoxic condition was taken. Thereafter, the normoxic buffer solution was switched to the hypoxic buffer solution, H_2_O_2_ or it was kept constant. Raman spectra were taken continuously, each with a shutter time of 2 min. After four minutes, the oxygen content was considered as hypoxic and a final spectrum termed HOX was taken. Then the buffer solution was switched to NOX again. After four more minutes, the recovery state was reached, a final Raman spectrum was taken and the experiment was terminated. Each experiment was carried out on different cells, grown on distinct glass bottom dishes (n = 36). A summary of the experimental plan and measurements that were used for analysis can be seen in Table 1.

### 2.5. Oxygen Sensing

The partial Oxygen pressure (pO_2_) inside the measurement chamber was continuously recorded using an optical needle-type oxygen sensor in combination with the corresponding Oxygen Logger-Software (Firesting, Pyro Science, Aachen, Germany) and analyzed using IGOR Pro 6.37 (Wavemetrics, Lake Oswego, OR, USA).

### 2.6. Data Analysis

To visualize the acquired Raman spectra for each set of experiments, the spectra were processed with the following steps:

1. Removal of cosmic rays—The second derivative was used to identify cosmic rays [21], which were removed and then inpainted using a local Savitzky-Golay filter (order 3, window size 41).
2. Signal Reconstruction—Piecewise average of least square fitted polynomials (order 3, window size 50 cm^−1^ for fitting, trimmed to 25 cm^−1^ in reconstruction to reduce the influence of possible corner effects).
3. Background Reduction—The MATLAB R2018a function msbackadj was used to remove a minimal common background for each reconstructed time series (step size 25 cm^−1^ and window size 25 cm^−1^). The background reduction was adjusted to make sure that no values in the estimated background could exceed the counts in the reconstruction.
4. Normalization with L2-norm.

Further, *t*-tests between the NOX and HOX measurements were performed on the semi-treated data—after cosmic ray removal and normalization—in the seven regions defined in Table 2. No background reduction was necessary here since the constant background does not affect the *t*-test. The *t*-test is often used in Raman measurements and it is able to handle small sample sizes [22]. This property makes the *t*-test suitable for the present study, since the number of samples from each experiment ranges from n = 4 to n = 11 (see Table 1).

## 3. Results

### 3.1. Oxygen Sensing

**Figure 4:**
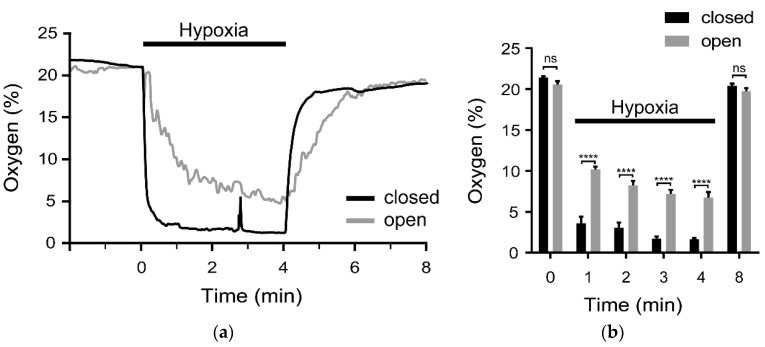
(**a**) Oxygen content inside the open (grey trace) and the closed, microfluidic system (black trace) upon switching from normoxic to hypoxic solution (indicated by the black bar). O_2_-curves are superimposed for better comparison and representative for n = 6 each; (**b**) Statistical analysis of experiments depicted in panel (**a**). Under hypoxic conditions, the O_2_-content was significantly lower in the closed, microfluidic system (black), compared to the conventional open system (grey). The 2-way ANOVA with Tukey’s multiple comparison test; ns: not significant *p* ≥ 0.5, **** *p* ≤ 0.0001.

The O_2_ content was continuously recorded via an optical O_2_-sensor that was placed inside the measurement chamber. One minute after switching to hypoxic buffer, the O_2_-content within the open system had dropped from 20.6 ± 0.4% to 10.2 ± 0.4%. Over the same time period, significantly lower hypoxic conditions of 3.6 ± 0.8% O_2_ were achieved in the closed microfluidic system (Figure 4; n = 6; *p* ≤ 0.001). Moreover, even after hypoxia application of 4 min, the oxygen content in the open system did not reach the same level as in the closed system (open system: 6.7 + 0.7% O_2_; closed system: 1.6 + 0.2% O_2_; n = 6; *p* ≤ 0.001.)

In addition to the slow and blunted drop in O_2_-content, the hypoxic condition in the open system was very unstable and characterized by multiple fluctuations (compare Figure 4a). By contrast, a stable hypoxic condition was achieved in the new, closed system (compare Figure 4b). Note, a spike in the oxygen level is visible prior to the 3 min mark.

### 3.2. Raman Spectral Investigation and Analysis

**Figure 5:**
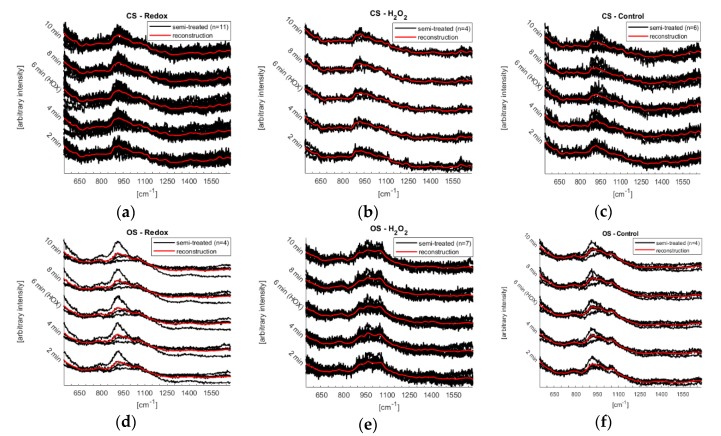
Reconstruction of the Raman response from measurements on PASMCs—Redox, H_2_O_2_ and control. (**a**–**c**) are from the closed microfluidic system (CS) and (**d**–**f**) are from the open system (OS). The 2 min mark is the initial stage, 6 min mark is the HOX-spectra followed by recovery. The reconstructed curve (red) is placed on top of the semi-treated data (black).

Figure 5 shows the results from the reconstructed Raman response with the cosmic rays removed. The reconstructed spectrum (red trace) is plotted along with the semi-treated data (black). They were used for *t*-tests.

The semi-treated data had different signal to noise levels in all cases (Figure 5).

**Figure 6:**
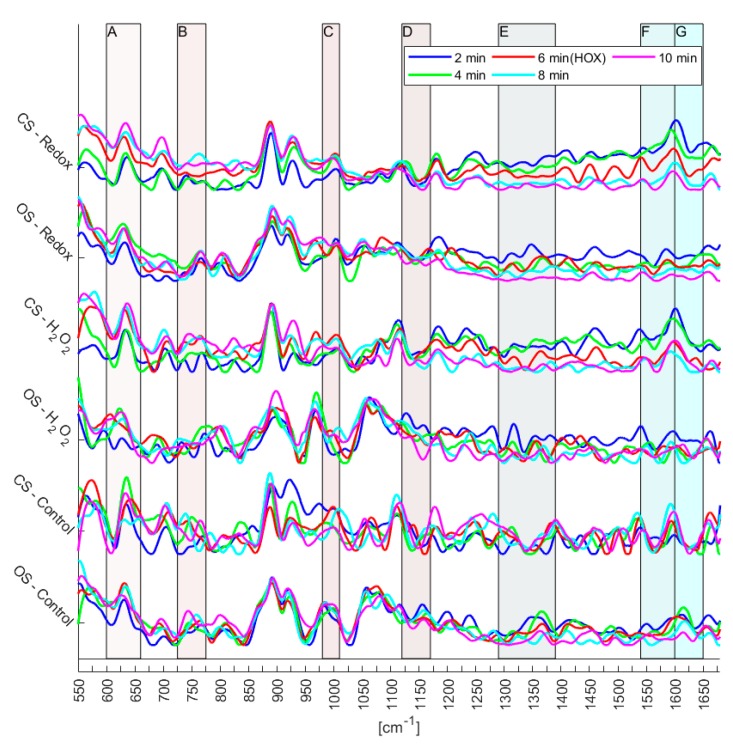
Stacked processed time series of Raman spectra from each experiment—Redox, H_2_O_2_ and control—in the closed system (CS) and open system (OS).

Figure 6 shows the Raman spectra after having removed a mathematically based background—not a background spectrum from the microfluidic system itself. The reason for this procedure is to be able to visualize the influence of the two systems (open vs. closed) on the measurements. Further, a subtraction of a background spectrum would add to the noise while not adding to the ability of detecting changes in Raman peaks. Along with the Raman spectra the regions that were used for *t*-test are shown, remember that the *t*-tests were not based upon the data shown in Figure 6 but rather on the semi-treated data shown in Figure 5. See Table 2 for a summary of the domain of all regions and which biomarkers are known to reside within each region.

The variations of the spectra—especially in the regions of the biomarkers (A–G)—are much more pronounced in the redox and the H_2_O_2_ experiments than in the control measurements (see Figure 6).

**Figure 7:**
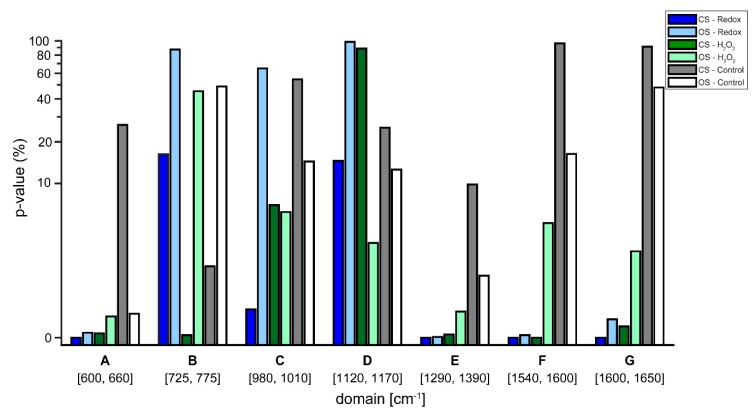
*p*-Values from closed system (CS) and open system (OS) for the spectral difference between normoxic and hypoxic conditions. *p*-values were computed in the seven domains (**A**–**G**) that contain Raman lines of the biomarkers (cytochromes, FeS or NADH).

Figure 7 shows *p*-values from *t*-tests between normoxic and hypoxic conditions on the semi treated data shown in Figure 5 and the seven regions specified in Table 2. Note that the *p*-value is always smaller in the redox measurements. In the control measurements, however, the *p*-value fluctuates.

## 4. Discussion

The aim of the present study was to develop a customized system that allows for real time-investigations of single PASMCs’ redox state in response to tightly controlled O_2_-concentrations—while providing the opportunity to simultaneously measure Kv-channel activity and membrane potential of the same cell via the patch-clamp technique.

This goal was achieved by designing a gas-tight microfluidic system enabling Raman spectroscopic investigation that additionally included a gas-tight port for a patch-pipette and one for the respective reference electrode. Due to a purpose-built latex glove, the patch-pipette can be precisely moved in 3D to be attached to PASMCs that are adhered to the bottom of a common culture dish. Moreover, once fixed to the system, all components needed for the experiment (reference electrode, O_2_-sensor and tubes for in- and outflow) can stay permanently attached to the system during experiment. The assembled microfluidic system was simply put upon the cell culture dish providing an unambiguous reduction in experiment time and simplification in handling. Furthermore, the PASMCs did not have to be trypsinated and/or transferred prior to experiment. Due to this, our new microfluidic system connotes (a) significantly less stress for the cells and (b) the possibility to measure adhered PASMCs (and other cell types).

To investigate the hypoxia-application, the oxygen content was measured by an optical oxygen sensor. The results show that the new microfluidic system has major advantages. First, after 1 min the O_2_-content was significantly lower in the new system compared to the conventional open one. Second, the value of less than 4% O_2_ proved to be stable over time, while massive fluctuations in O_2_-content occurred in the open system. Therefore, future experiments using the new gas-tight system can be carried out in shorter experimental times (HOX-spectra can be taken after 1 min)—which means less stress for the PASMCs due to the reduced laser exposure time. Note, spikes in the oxygen level can appear randomly (see Figure 4a) which may influence the Raman spectrum. However, by having control over when the spikes appear, they should not impose on the total reliability of the data since those spectra, thanks to the simultaneous measurements, can be excluded.

The microfluidic system was further tested by performing Raman spectroscopy on single murine PASMCs. The laser beam was defocused to 20 μm to get an average over the whole intracellular components of the single PASMC. Together with the low power chosen (0.6 mW) the experimental conditions resulted in a high signal to noise ratio, see Figure 5. Furthermore, the number of experiments does not yet allow the identification of reversible changes of the mitochondrial biomarkers. However, since certain trends can be observed (Figure 6), first conclusions can be drawn: Raman spectra of the control experiments showed a continuous fluctuation while the fluidic system was kept at a flow of a steady normoxic O_2_-content during the whole experiment. However, the control measurements were fluctuating around a constant baseline, which was not the case for the Redox and H_2_O_2_ experiments (compare Figure 6). This fluctuation of the Raman signal seems to be in agreement with the study of Almohammedi et al. [19]. In addition, for Redox as well as H_2_O_2_, a shift in the entire spectra was observed for both the closed and the open microfluidic system. Although this response appeared in both systems, it was less pronounced in the open system. There, they rather resembled the general fluctuations that—according to the control experiments—seem to exist intrinsically in PASMCs.

Lastly, *t*-tests in the regions of the defined biomarkers presented in Table 2 were performed on the semi-treated Raman spectra. The aim was to acquire quantitative measures on the likelihood, if the observed changes resulted from noise or from the single cell redox reactions (see Figure 7). The *t*-tests confirmed the observations from the treated Raman signal (see Figure 6). Regarding the control experiments, the *t*-test generally resulted in a high likelihood of changes being due to noise for both, the closed and the open system. In some cases, the changes were significant, which implies that the system is in a general state of fluctuation—which is to be expected for a living system. Interestingly, the level of noise was more pronounced in the closed system (see Figure 5), a finding that needs further investigation. Regarding the Redox and H_2_O_2_ experiments, it is evident that *p*-values that resulted from changes relating normoxic to hypoxic conditions showed an increased likelihood of being a true change for the closed system compared to the open system. To emphasize, as shown in Figure 4, the closed system created stable hypoxic conditions, which hints that the changes in the processed spectra (see Figure 6) are due to the changes from normoxia to hypoxia. Only in two cases, the open system appeared to trump the closed system, these occurred in the regions C (980–1100 cm^−1^) and D (1120–1170 cm^−1^) for the H_2_O_2_ experiments. For region C, the reported *p*-values were 6.05% for the open system and 6.87% for the closed system, a difference of only 0.82% in favor of the open system. In region D, the *p*-value from the closed system was 88.73%, indicating a high likelihood of changes being due to noise, however, Figure 6 reveals that in region D of the spectra there appears to be little actual change between the various time steps, regardless of experiment.

At this point, it seems evident that the closed microfluidic system improves the ability to identify Raman spectral changes caused by redox state in PASMCs. It has to be mentioned that the material of the microfluidic system was polycarbonate, having Raman peaks at 644 cm^−1^, 741 cm^−1^, 761 cm^−1^, 1013 cm^−1^, 1121 cm^−1^, 1190 cm^−1^, 1297 cm^−1^, 1320 cm^−1^, 1372 cm^−1^, 1396 cm^−1^, 1549 cm^−1^, 1611 cm^−1^ [23] that could interfere with the defined biomarkers (Table 2). Since the Raman signal from the polycarbonate is constant, it should not contribute to the redox spectral changes and hence it should not interfere with the results from the *t*-test. However, the polycarbonate background can overpower the Raman peaks from the biomarkers. In this study, the background from neither the polycarbonate nor the glass slide were removed for signal analysis, since this would have reduced the signal-to-noise ratio and does not contribute to the recognition of large scale changes to the Raman spectra. The material of the microfluidic system and Raman spectroscopic acquisition parameters will be optimized for further experiments.

Overall the new design of the gas-tight microfluidic system gives the extraordinary possibility to simultaneously perform (a) single-cell Raman spectroscopic studies to investigate mitochondrial biomarkers (redox state) and (b) patch-clamp experiments to study the Kv-channel activity under normoxic/hypoxic conditions. This new method might elucidate the molecular mechanism of how hypoxia—or the subsequent change in the cellular redox state—inhibits Kv-channels and how this process may initiate HPV.

## 5. Conclusions

A gas-tight microfluidic system with the option to simultaneously carry out Raman spectroscopic measurements and patch-clamp experiments on the single cell level was developed. The system was designed to easily fit onto a common culture dish having a glass bottom for microscopic investigations. The fragile patch-clamp pipette could be inserted via a flexible latex glove that enabled 3D movement of the pipette. In this study, the microfluidic system was tested regarding the oxygen content and the possibility to perform Raman spectroscopic investigations on single cells. The results show that the microfluidic system generated a stable and lower O_2_ content already after one minute compared to the commonly used experimental setup, where the culture dish is exposed to open air while flushed with oxygen free buffer. This significantly reduces the stress on the PASMCs. The Raman spectroscopic time-series showed that the spectral changes were much more pronounced in the new gas-tight microfluidic system compared to the open system. The new design of the flow system shows great promise for future single cells investigation to elucidate the process underlying HPV by performing Raman spectroscopy and patch-clamp simultaneously under truly hypoxic conditions.

## Figures and Tables

**Table 1:** Number of time series of Raman spectra for analysis.

Type of Experiment	Number of Experiments Open System	Number of Experiments Closed System
Redox	4	11
H_2_O_2_	7	4
Control	4	6

**Table 2:** Regions with known biomarkers used in *t*-test.

Region	Domain (cm^−1^)	Peak (cm^−1^)	Biomarker
A	600–660	604	cyt b [16], cyt c, cyt c1 [16,17], deoxy-cyt c [18]
		650	FeS [16]
B	725–775	750	cyt b, cyt c, cyt c1 [16,17], deoxy-Mb [18]
C	980–1010	991, 1000	NADH [16]
D	1120–1170	1127, 1167	cyt b, cyt c, cyt c1 [16,17]
E	1290–1390	1300–1303	cyt b [17], deoxy-cyt b, oxy-cyt b [18]
		1305	cyt b, cyt c, cyt c1 [17]
		1313	cyt c [17], deoxy-cyt c, oxy-cyt c [18]
		1337	cyt b [17], deoxy-cyt b, oxy-cyt b [18]
		1356–1358	deoxy-Mb [17,19]
		1372–1377	oxy-cyt c [19], oxy-Mb [17,19]
F	1540–1600	1545–1548	deoxy-Mb, deoxy-cyt c [19]
		1556	deoxy-Mb, oxy-Mb b [17]
		1563–1565	oxy-cyt c, deoxy-Mb [19]
		1582–1587	cyt b, cyt c, cyt c1 [16], deoxy-cyt c, oxy-cyt c, deoxy-Mb, oxy-Mb [19]
G	1600–1650	1606–1608	deoxy-Mb [16,19]
		1622	deoxy-cyt [19]
		1638	cyt c [16], oxy-cyt [18], oxy-cyt c [18,19]
		1640–1642	oxy-Mb [17,19]
